# k mdiff, large-scale and user-friendly differential *k*-mer analyses

**DOI:** 10.1093/bioinformatics/btac689

**Published:** 2022-10-31

**Authors:** Téo Lemane, Rayan Chikhi, Pierre Peterlongo

**Affiliations:** Univ. Rennes, Inria, CNRS, IRISA - UMR 6074, Rennes, F-35000 France; Institut Pasteur, Université Paris Cité, Sequence Bioinformatics, Paris, F-75015, France; Univ. Rennes, Inria, CNRS, IRISA - UMR 6074, Rennes, F-35000 France

## Abstract

**Summary:**

Genome wide association studies elucidate links between genotypes and phenotypes. Recent studies point out the interest of conducting such experiments using *k*-mers as the base signal instead of single-nucleotide polymorphisms. We propose a tool, kmdiff, that performs differential *k*-mer analyses on large sequencing cohorts in an order of magnitude less time and memory than previously possible.

**Availabilityand implementation:**

https://github.com/tlemane/kmdiff

**Supplementary information:**

Supplementary data are available at *Bioinformatics* online.

## 1 Introduction

Genome wide association studies (GWAS) determine links between genotypes, i.e. genomic variants and phenotypes such as diseases. GWAS are generally performed either by genotyping known variants using micro-arrays or by mapping vast amount of sequenced data to reference genomes. In both cases, the data are biased and incomplete as genotypes are a predefined set of single-nucleotide polymorphisms (SNPs), with respect to a particular reference genome. Parts of individual genomes from a population which are absent from this reference, or which do not align to it, are simply omitted. Recent approaches (Mehrab *et al.*, 2021; Rahman *et al.*, 2018; Voichek and Weigel, 2020) propose to overcome those limitations by directly comparing raw sequencing data without resorting to a reference genome. Despite being of fundamental interest these tools are clearly under-exploited, likely because of important practical limitations: a high expertise required for installing and running the tools and more importantly because of prohibitive computational requirements even for only dozens of individuals.

Here, we present kmdiff, a new tool that performs large reference-free GWAS experiments using *k*-mers. kmdiff is based on state-of-the-art statistical models described in HAWK (Rahman *et al.*, 2018), which detect *k*-mers with significantly different frequencies between two cohorts, taking into account population stratification. The main novelties offered by kmdiff are its usability (user-friendly installation and usage) and its performance, being up to 16× faster than HAWK and using 9× less RAM and nearly 3× less disk. These features enable kmdiff to compare dozens of human whole-genome sequencing experiments in a few hours using reasonable hardware resources.

## 2 Methods

### 2.1 Kmdiff pipeline

**Fig. 1.**
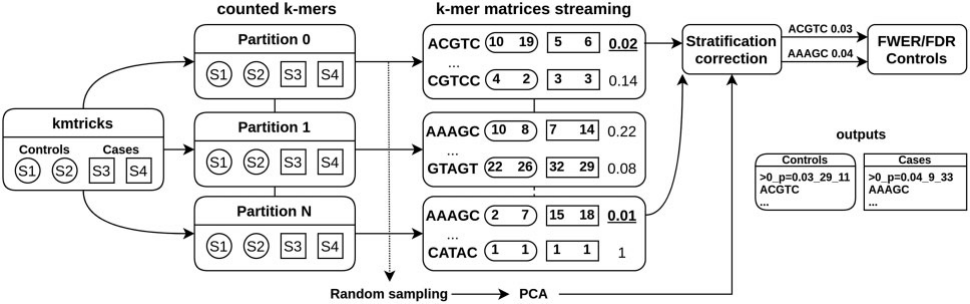
kmdiff pipeline overview on two cohorts composed of two samples: S1 and S2 for controls in round boxes and S3 and S4 for cases in square boxes. (A) First stage corresponds to partitioned \kmer counting with kmtricks. (B) Matrix streaming process during which k-mers are tested for significance and sampled to contribute to the PCA. (C) Significant P-values are corrected to account for the population stratification and are then screened by common controlling procedures. The k-mers ACGTC and AAAGC are over-represented in controls and cases, respectively

For the statistical part, kmdiff follows HAWK both in terms of *k*-mer detection and population stratification correction. Each *k*-mer is tested for significant association with either cohort using a likelihood ratio test, which assumes that *k*-mers are Poisson-distributed. To take into account the population stratification and thus to compute corrected *P*-values, a random sample of *k*-mers (<1/100th of total) are used to infer a stratification using the Eigenstrat software (Patterson *et al.*, 2006; Price *et al.*, 2006; Rahman *et al.*, 2018). Finally, *P*-values are adjusted for multiple tests (Salkind, 2006) using Bonferroni correction (though Benjamini–Hochberg can also be used).

kmdiff deviates from HAWK in the *k*-mer counting part. HAWK counts *k*-mers of each sample before loading and testing batches of them using a hash table. The *k*-mer abundance tables are obtained using a slightly modified version of Jellyfish (Marçais and Kingsford, 2011) bundled with the tool. Instead, kmdiff constructs a *k*-mer matrix, i.e. an abundance matrix with *k*-mers in rows and samples in columns. For efficiency reasons and to limit drastically the memory usage, this matrix is not represented as a whole but sub-matrices are streamed in parallel using kmtricks (Lemane *et al.*, 2022). An overview of the procedure is shown in Figure 1.

### 2.2 Implementation

kmdiff is a well-documented and user-friendly command line tool implemented in C++. It extensively uses the kmtricks tools and APIs for efficient *k*-mer matrix construction. It also supports C++ plugins to easily prototyping new stream-friendly models while keeping the pipeline efficiency. Sources and documentation are available at https://github.com/tlemane/kmdiff.

## 3 Results

We compare the performance of kmdiff with the state-of-the-art tool HAWK and demonstrate the ability of kmdiff to be more scalable while producing an equivalent output. We present medium and large-scale experiments adapted from Rahman *et al.* (2018), respectively on bacterial and human data. Extended results, together with the benchmark environment and resources description are available as a supplement (see Supplementary Section S1).

We also compared the computational performances of kmdiff to kmerGWAS (Voichek and Weigel, 2020), but not the quality of results, as kmerGWAS uses a different statistical model which does not compare two cohorts but instead considers phenotypes as continuous real values. Because of the high memory usage of kmerGWAS, results are limited to the bacterial dataset (see Supplementary Section S1.2).

### 3.1 Ampicillin resistance

This dataset consists of sequencing data from 241 strains of *Escherichia coli* from Earle *et al.* (2016). Among them 189 are resistant to ampicillin and 52 are sensitive. On this dataset, kmdiff is 6× faster than HAWK and reduces memory and disk usage by 8× and 4.5×, respectively. The difference in memory usage is explained by the use of kmtricks, a disk-based counting algorithm. For the disk usage, the difference is due to the compressed representation of counted *k*-mers. The *k*-mers found are exactly the same for both tools: 13196814 over-represented *k*-mers occur in cases, and 16804587 in controls. After population stratification, due to stochasticity, results differ: 4542 (for HAWK) and 4591 (for kmdiff) *k*-mers from controls pass significance filters. The difference can be explained by imprecise floating-point arithmetics and non-deterministic sub-sampling during population stratification correction. Thus, some *k*-mers with *P*-values close the significance threshold may not be found by both tools. In this experiment, 98% of *k*-mers found by HAWK are also found by kmdiff. The distribution of the significant *P*-values reported by both two tools is available in the Supplementary Material.

### 3.2 Human cohorts

**Fig. 2.**
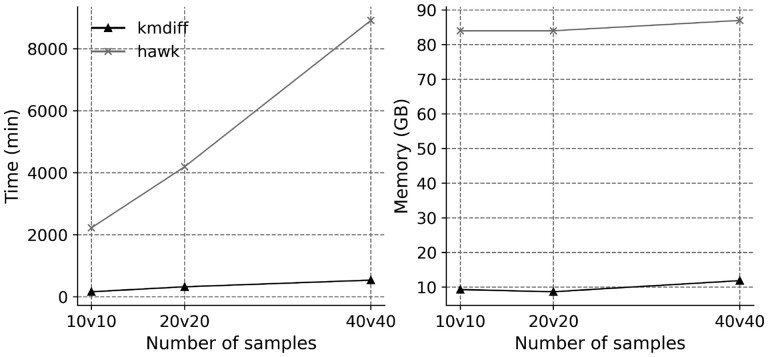
Scalability of HAWK and kmdiff on human cohorts. Both tools support multi-threading and were executed using 20 threads. kmdiff reduces computation times by 13–16× and memory usage by 8×

To illustrate the scalability of kmdiff, we compared it to HAWK on several datasets of different sizes from the 1000 Genome project (The 1000 Genomes Project Consortium, 2015). We used whole-genome sequencing from two populations, TSI (Toscani in Italia) and (Yoruba in Ibadan, Nigeria), to build benchmark datasets composed of 20, 40 and 80 individuals. As shown in the Figure 2, kmdiff offers a better scalability than HAWK being at least 13 times faster while using significantly less memory and disk.

## 4 Conclusion

kmdiff enables differential *k*-mer analysis over large cohorts of sequencing data. It provides results that are equivalent to the state-of-the-art tool HAWK, but it is an order of magnitude more efficient. It additionally has the advantage of being easy to install and use. Finally, kmdiff is designed to allow simple addition of new streaming-friendly models making future updates possible while maintaining the pipeline efficiency.

## Supplementary Material

btac689_Supplementary_Data
