# Supplementary material for: k mdiff, large-scale and user-friendly differential k-mer analyses
Source: Bioinformatics. 2022 Oct 31;38(24):5443–5. doi: 10.1093/bioinformatics/btac689 (PMC9750116; doi:10.1093/bioinformatics/btac689)
Supplement: btac689_Supplementary_Data [file btac689_supplementary_data.pdf]

Supplementary

Complete list of accession numbers, files and command lines are available on the github companion (`https://github.com/tlemane/kmdiff-experiments`).

S1 Benchmarks

S1.1 Environment

All experiments were run on the GenOuest platform on a node with 2x24-cores Xeon Gold 5220R 2,20 GHz with 128 GB of memory and a filesystem allowing 900 MB/s and 290 MB/s sequential read/write. The experiments was performed using `kmdiff` v1.0.0, `HAWK` v1.7.0 and `kmerGWAS` v0.2. All tools support multi-threading and were executed using 20 threads.

S1.2 Results

Ampicilin 189v52	Time (min)			Memory (GB)			Disk (GB)		
	cnt	dff	sum	cnt	dff	max	cnt	dff	max
`HAWK`	103	171	274	6.1	8.1	8.1	28.7	67.1	67.1
`kmerGWAS`	89	15	104	3.5	1.9	3.5	10.3	3.4	**10.3**
`kmdiff`	11	36	**47**	2.7	2.7	**2.7**	14.8	5.7	14.8

Human 10vs10	Time (min)			Memory (GB)			Disk (GB)		
	cnt	dff	sum	cnt	dff	max	cnt	dff	max
`HAWK`	2040	186	2226	84	21.2	84	1024	20.2	1024
`kmdiff`	129	37	**166**	9.3	2.4	**9.3**	380	4.7	**380**

Human 20vs20	Time (min)			Memory (GB)			Disk (GB)		
	cnt	dff	sum	cnt	dff	max	cnt	dff	max
`HAWK`	3916	277	4193	84	28.5	84	2016	32.7	2016
`kmdiff`	241	83	**324**	8.7	3.26	**8.7**	726	9.34	**726**

Human 40vs40	Time (min)			Memory (GB)			Disk (GB)		
	cnt	dff	sum	cnt	dff	max	cnt	dff	max
`HAWK`	8319	592	8911	87	48.6	87	3914	93.5	3914
`kmdiff`	418	122	**540**	11.9	6.2	**11.9**	1455	49.4	**1455**

Table S1. Benchmarks of `HAWK`, `kmdiff` and `kmerGWAS` on different scale datasets. We break down the time, memory and disk usage columns into the resources needed for the two analysis steps: `cnt`. the k-mer counting step (which for `HAWK`, includes the sorting of k-mers, which is not required for `kmdiff`) and `dff`. the k-mer association step, including k-mers aggregation, statistical tests and population stratification correction. `kmerGWAS` results are not available on human datasets because it requires an amount of memory (> 128 GB) that exceeds the capacity of our benchmark machine.

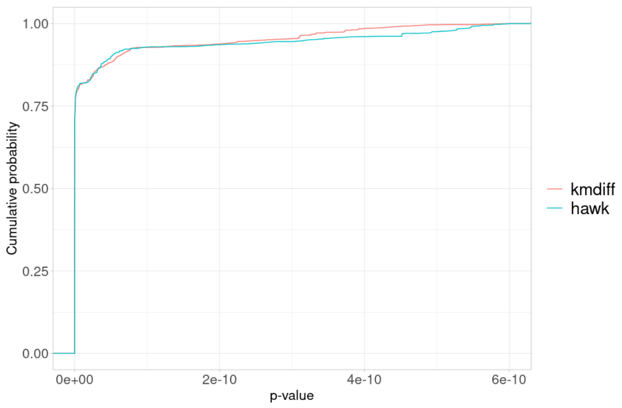

Fig. S1: Cumulative distribution function of the p-values of the significant k-mers reported by both `kmdiff` and `HAWK` on the ampicilin resistance dataset.
